# Oxidative stress and docosahexaenoic acid injury lead to increased necroptosis and ferroptosis in retinal pigment epithelium

**DOI:** 10.1038/s41598-023-47721-5

**Published:** 2023-11-30

**Authors:** Almar Neiteler, Anwar A. Palakkan, Kevin M. Gallagher, James A. Ross

**Affiliations:** 1 Tissue Injury and Repair Group, University of Edinburgh, Chancellor’s Building, 49 Little France Crescent, Edinburgh, EH16 4SB UK; 2 Immunology and Stem Cell Biology, Aravind Medical Research Foundation, Anna Nagar, Madurai, 625020 India

**Keywords:** Necroptosis, Mechanisms of disease, Macular degeneration, Fatty acids, Risk factors

## Abstract

Age-related macular degeneration (AMD) is a complex disease caused by different genetic and environmental risk factors leading to loss of cells in the central part of the retina. Oxidative stress appears to be an important environmental risk factor that contributes to both the initiation and progression of AMD. Retinal pigment epithelium (RPE) plays an important role in regulating oxidative stress in the retina and is one of the main retinal cell types affected in AMD. A main function of RPE is to phagocytose photoreceptor outer segments (POS) which are rich in the polyunsaturated fatty acid (PUFA) docosahexaenoic acid (DHA), making this cell type potentially more susceptible to oxidative stress-induced lipid peroxidation which can lead to cell death. RPE is known to undergo necrotic cell death in response to oxidative stress. The aim of this study was to determine if DHA in POS can increase oxidative damage to RPE. It was found that RPE undergo increased lipid peroxidation and decreased cell viability when stressed with hydrogen peroxide in combination with DHA or POS. H_2_O_2_-induced oxidative stress was found to cause both ferroptosis and necroptosis. However, the ferroptosis regulator acyl-CoA synthetase long-chain family member 4 (ACSL4) was found to be downregulated in RPE exposed to H_2_O_2_ and this effect was exacerbated when the RPE cells were simultaneously treated with DHA. Together, these results show a response of RPE when stressed which will likely be overwhelmed under disease conditions such as AMD resulting in cell death.

## Introduction

Age-related macular degeneration (AMD) is the most common cause of visual loss in the elderly in developed countries^1–3^. Oxidative stress is commonly attributed to age-associated pathology in many diseases with damage from oxidative stress accumulating during ageing^4^. In addition, the antioxidant capacity diminishes and the efficiency of repair systems is compromised with age^5,6^. Oxidative stress also has an important role in the initiation and progression of AMD^7,8^. Moreover, treatments with antioxidants, such as vitamin C, vitamin E, β-carotene, and zinc, have been found to ameliorate AMD^7^. Various stimuli can induce different forms of oxidative stress in the eye^9^. Proportionally compared to the rest of the body, the retina consumes the most oxygen and therefore has several adaptations to deal with oxidative stress^10^. Dysregulation of pathways regulating oxidative stress are therefore likely to play a role in AMD pathology.

Retinal pigment epithelium (RPE) cell death is characteristic of late stage AMD^11^. The RPE has many important functions in the retina, including light absorption, transepithelial transport, buffers ions in the subretinal space, participates in the visual cycle, phagocytoses photoreceptor outer segments (POS), and secretes important factors to neighbouring cells^12^. In the retina, the RPE cells are continuously exposed to oxidative stress as RPE metabolises and recycles POS^12,13^. RPE cells also have large oxygen fluxes near the plasma membrane^12,14^ and are exposed to light^10,12^. Moreover, aged RPE have been found to have a reduced metabolic capacity, leading to increased susceptibility to oxidative stress^15^. Therefore, RPE need to be well adapted to handle oxidative stress.

An important function of RPE is to phagocytose POS^16,17^. POS are enriched with PUFAs, to the point that POS possess the highest relative concentration of PUFAs compared to all other body tissues^18^. The high amount of double bonds in PUFAs make them susceptible to lipid peroxidation by reactive oxygen species (ROS). Docosahexaenoic acid (DHA; 22:6) is an omega-3 PUFA that is relatively rare in human tissue. However, DHA accounts for 60% of the PUFAs in the retina, with its highest concentration in POS^18^. DHA is an essential structural component of the cell membrane in POS^19^ and DHA is known to be able to enhance RPE survival^20^. However, DHA is also the most oxidizable fatty acid in humans^18^. RPE are constantly exposed to high concentrations of DHA through phagocytosis of POS and through transport of DHA from the choriocapillaris to the photoreceptors for photoreceptor membrane biogenesis^21^. Oxidative stress can therefore potentially cause lipid peroxidation of DHA in RPE, leading to RPE damage and possibly cell death.

Oxidative stress caused by hydrogen peroxide (H_2_O_2_) has been shown to lead to necroptosis, and not apoptosis, in RPE^22^. Necroptosis is a regulated form of necrosis^23^. Moreover, oxidative stress induced by *tert*-butyl hydroperoxide (tBHP) or by glutathione depletion were recently shown to cause ferroptosis in human cells, including RPE^24–26^. Ferroptosis is an iron- and PUFA-dependent form of regulated cell death, which is distinct from apoptosis and necrosis^27^. DHA is also known to be able to induce ferroptosis^28^. However, it is not yet known if DHA can contribute to RPE cell death through ferroptosis during oxidative stress.

Dysregulation of antioxidant defensive mechanisms in the retina of AMD patients is not well understood. Equally, it is not well understood how dysregulation of lipid metabolism under such conditions contributes to RPE cell death and ultimately leads to vision loss.

Here, we show that increased intracellular POS or DHA concentration can increase RPE susceptibility to oxidative stress injury, leading to both ferroptosis and necroptosis of RPE cells.

## Materials and methods

### Cell culture

ARPE-19 (ATCC, Manassas, VA) and hTERT RPE-1 (ATCC) cell lines were cultured and maintained in DMEM/F12 (Gibco, Loughborough, UK) supplemented with 10% foetal calf serum (FCS; Gibco) and l-glutamine (Gibco) at 37 °C and 5% CO_2_. For experiments, cells were split and seeded in DMEM/F12 supplemented with 1% FCS and l-glutamine unless otherwise stated.

### Fatty acid-BSA conjugation

DHA (Sigma-Aldrich, Dorset, UK) and palmitic acid (PA; Sigma-Aldrich) were conjugated to fatty acid-free bovine serum albumin (BSA; Sigma-Aldrich) as previously described^29^, but with some modifications. Briefly, DHA and PA were dissolved in 50% ethanol to 150 mM at 70 °C and 90 °C respectively. The dissolved fatty acids were then immediately diluted, using prewarmed tips, in prewarmed 10% fatty acid-free BSA solution to 7.5 mM whilst stirring. This was incubated at 37 °C for 1 h to achieve BSA-fatty acid conjugation. The conjugated BSA-fatty acids were 0.22 µm filter-sterilised, aliquoted, and stored at − 20 °C. Conjugated BSA-fatty acids were used in cell culture medium at a final concentration of 500 µM fatty acid and 0.67% BSA unless otherwise stated.

### POS-FITC conjugation

Bovine rod POS (InVision BioResources, Seattle, USA) were prepared and conjugated with fluorescein-5-Isothiocyanate (FITC; Invitrogen) as previously described^30^, with some modifications. POS were washed three times with and resuspended in wash solution, consisting of 10% sucrose, 20 mM sodium phosphate buffer pH 7.2, and 5 mM taurine. To obtain a 2 mg/ml working solution of FITC, 10 mg of FITC was dissolved in 0.1 M sodium-carbonate buffer pH 9.5. To conjugate POS with FITC, 1.5 ml of FITC working solution was added to 5 ml of POS suspension and incubated at 21 °C for 1 h whilst rotating in the dark. The POS-FITC conjugate was then washed twice with and resuspended in DMEM with 2.5% sucrose.

### Inhibitor treatment, oxidative stress treatment, and immunocytochemistry

RPE cell lines were seeded at 7.5 × 10^3^ cells/well in a 96-well plate. The following day the cells were pre-treated for 24 h with 40 µM Z-VAD-FMK (Merck Millipore, Nottingham, UK), 40 µM Necrostatin-1 (Nec-1; Cayman Chemical, Ann Arbor, MI), 1 µM GSK2791840B (GSK’840B; GlaxoSmithKline, Brentford, UK), or 5 µM Ferrostatin-1 (Fer-1; Sigma-Aldrich). Pre-treatment with 500 µM deferoxamine (DFO; Sigma-Aldrich) was 1 h before injury.

Medium with inhibitors was transferred to a new plate and kept at 37 °C and 5% CO_2_. The cells were subsequently stained with 3 µM (4,4-difluoro-5-(4-phenyl-1,3-butadienyl)-4-bora-3a,4a-diaza-s-indacene-3-undecanoic acid; Invitrogen) in culture medium for 1 h at 37 °C and 5% CO_2_ to assess lipid peroxidation in RPE cell line membranes. C11-BODIPY^581/591^ incorporates into membranes and the excitation and emission fluorescence spectra of this lipid analogue both shift to shorter wavelengths when oxidised^31^. The cells were washed once with PBS and the medium with inhibitors was transferred back to the cells.

The cells were then treated with a final concentration of 500 µM H_2_O_2_ (Sigma-Aldrich) for 6 h or 500 µM DHA or 50 POS-FITC/cell for 18 h at 37 °C and 5% CO_2_.

After treatment, the cells were washed with PBS and subsequently stained with 6 µg/ml Hoechst 33342 (Sigma-Aldrich). The cells were then fixed with prewarmed 4% PFA for 10 min at 21 °C and washed with PBS.

For pMLKL and PI staining, the cells were washed with PBS after H_2_O_2_ treatment and subsequently stained with 3 µg/ml PI (Sigma-Aldrich) for 20 min at 21 °C. The cells were then fixed with prewarmed 4% PFA for 10 min and permeabilised with 0.1% Triton™ X-100 (Sigma-Aldrich) for 15 min at 21 °C. The cells were then incubated with 1:100 phospho S358 MLKL antibody (Abcam, Cambridge, UK; ab187091) in 3% goat serum for 1 h at 4 °C. After two washes with PBS, the cells were incubated with 1:250 Alexa Fluor 647 antibody (Molecular Probes; A21244) for 30 min at 21 °C covered from light. After another two washes with PBS, the nuclei were counterstained with 300 nM DAPI (Biotium, Fremont, CA) for 10 min at 21 °C covered from light, followed by another three washes with PBS.

Stained cells were imaged with a Leica DMi8 inverted microscope (Leica Microsystems, Milton Keynes, UK).

### Flow cytometry for lipid peroxidation

RPE cell lines were seeded and treated with inhibitors in 96-well plates as described above. Medium with inhibitors was transferred to a new plate and kept at 37 °C and 5% CO_2_. The cells were then incubated for 1 h at 37 °C and 5% CO_2_ with a final concentration of 3 µM C11-BODIPY^581/591^ in DMEM/F12 without phenol red (Gibco) and no FCS. The cells were washed once with PBS and the medium with inhibitors was transferred back to the cells. The cells were then treated with a final concentration of 500 µM H_2_O_2_ for 3 h at 37 °C and 5% CO_2_. After H_2_O_2_ treatment, the cells were washed once with PBS, trypsinised, and transferred to Eppendorf’s containing medium with 10% FCS to inactivate the trypsin. The cells were then washed once with flow buffer, resuspended in 250 µl of flow buffer containing a final concentration of 2.4 µM DRAQ7 (Biostatus, Shepshed, UK). The samples were kept on ice until analysis with a BD Accuri C6 flowcytometer (BD Biosciences, Franklin Lakes, NJ).

### Flow cytometry for Annexin V, PI, MLKL, and pMLKL

RPE cell lines were seeded and when stated treated with inhibitors in 96-well plates as described above. The cells were then treated with a final concentration of 500 µM H_2_O_2_ for 3 h at 37 °C and 5% CO_2_. After H_2_O_2_ treatment, the cells were washed once with PBS, trypsinised, and transferred to Eppendorf’s containing medium with 10% FCS to inactivate the trypsin.

For Annexin V and PI staining, the cells were stained using the Annexin V-FITC Apoptosis Detection Kit (Affymetrix, Santa Clara, CA) according to the manufacturer’s instructions.

For MLKL and pMLKL staining, the cells were fixed in prewarmed 4% paraformaldehyde for 10 min at room temperature. After washing with flow buffer, consisting of PBS with 1% BSA and 0.05% NaN_3_, the cells were permeabilised with 0.2% Triton X-100 for 5 min at room temperature. After washing with flow buffer the cells were incubated with 1:100 MLKL antibody (Abcam; ab184718) or 1:100 phospho S358 MLKL antibody (Abcam; ab187091)^32^ in 10% normal goat serum in flow buffer for 1 h on ice. After washing the cells were incubated with 1:250 Alexa Fluor 488 antibody (Cell Signaling Technology, Danvers, MA; 4412S) or 1:250 Alexa Fluor 647 antibody (Molecular Probes, Eugene, OR; A21244) for 30 min on ice. The cells were then washed once with flow buffer, resuspended in 250 µl of flow buffer. The samples were kept on ice until analysis with a BD Accuri C6 flowcytometer.

### Cell viability assay

RPE cell lines were seeded at 10^4^ cells per well in a 96-well plate. After 24 h, cells were then treated with 500 µM H_2_O_2_ or 500 µM BSA-PA or 500 µM BSA-DHA or H_2_O_2_ with BSA-PA or H_2_O_2_ with BSA-DHA for 6 or 18 h. Respective wells were pre-treated with 40 µM Nec-1 or 5 µM Fer-1 for 1 h before the treatment. Cells treated with 0.67% BSA and 0.17% ethanol were considered as the control (vector control). After treatment cellular viability was analysed using MTT. Briefly, cells were incubated with 0.5 mg/ml MTT (Sigma-Aldrich) for 4 h, and the formed formazan crystals were solubilised using 10% SDS-(0.01N)HCl solution. Absorbance at 570 nm and 690 nm was measured, and the 570/690 ratio was used for the comparison. The absorbance ratio of each group was compared to the control and expressed as a percentage.

### qPCR

RPE cell lines were seeded at 3 × 10^5^ cells/well in a 6-well plate. The following day the cells were treated with 300 µM H_2_O_2_ and 300 µM BSA-DHA for 24 h. RNA was extracted using TRIzol^®^ Reagent (Ambion, Waltham, MA) according to manufacturer’s instructions. cDNA was made using the High Capacity cDNA Reverse Transcription Kit (Applied Biosystems, Foster City, CA) according to manufacturer’s instructions and primers were designed by Primerdesign Ltd. (Southampton, UK), see Table S1 for sequences. Power SYBR^®^ Green PCR Master Mix (Applied Biosystems) was used in combination with a StepOnePlus™ Real-Time PCR System (Applied Biosystems). The relative gene expression data was analysed using the 2^−∆∆Ct^ method as described by Livak and Schmittgen^33,34^.

### Western blotting

RPE cell lines were seeded at 2.5 × 10^5^ cells/well in a 6-well plate. The following day the cells were treated with 500 µM H_2_O_2_, 500 µM BSA-PA, and 500 µM BSA-DHA for 6 h. The cells were then lysed in RIPA buffer supplemented with Complete Mini protease inhibitor cocktail (Sigma-Aldrich) and PhosSTOP phosphatase inhibitor (Roche, Basel, Switzerland) and passed through a 21-gauge needle (Sterican, Hessen, Germany) to shear the DNA. 20 µg of cell lysate in Laemmli sample buffer (Bio-Rad, Hercules, CA) supplemented with β-mercaptoethanol was heated to 98 °C for 5 min and subsequently loaded and ran on a 12% SDS-PAGE gel. The gel was then blotted onto a nitrocellulose membrane (Bio-Rad), blocked with TBS + 5% Marvel for 30 min at room temperature, and incubated with 1:500 ACSL4 antibody (Santa Cruz Biotechnology, Santa Cruz, CA; sc-271800) overnight at 4 °C. The following day the membrane was incubated with 1:4,000 HRP-conjugated secondary antibody (Merck Millipore; 12–349) for 30 min at room temperature. After incubation with Amersham ECL reagent (GE Healthcare, Little Chalfont, UK), an Amersham Hyperfilm (GE Healthcare) was exposed to the membrane. The same membrane was reprobed with 1:5,000 β-actin antibody (Abcam; ab6276) for 1 h at room temperature followed by 1:4,000 HRP-conjugated secondary antibody (Merck Millipore; 12-349) for 30 min at room temperature to check for loading control. Exposed films were developed in a Mi-5 X-ray developer machine (Jet Xray, London, UK).

### Statistics

Experiments were repeated at least three times. Statistical analysis was performed using Microsoft Excel version 1905 and GraphPad Prism 6 software. *P*-values of less than 0.05 were considered to be statistically significant.

## Results

### Increased lipid ROS in RPE after H_2_O_2_, DHA, or POS exposure and is inhibited by Fer-1 and DFO

RPE were exposed to H_2_O_2_ in order to investigate the role of apoptosis and necrosis in RPE cell death under oxidative stress conditions. H_2_O_2_-exposure did not induce apoptosis in RPE, although it did result in more necrotic cells (Fig. S1). This is in agreement with published findings^22^. However, other forms of cell death could also play a role in addition to necrosis.

**Figure 1:**
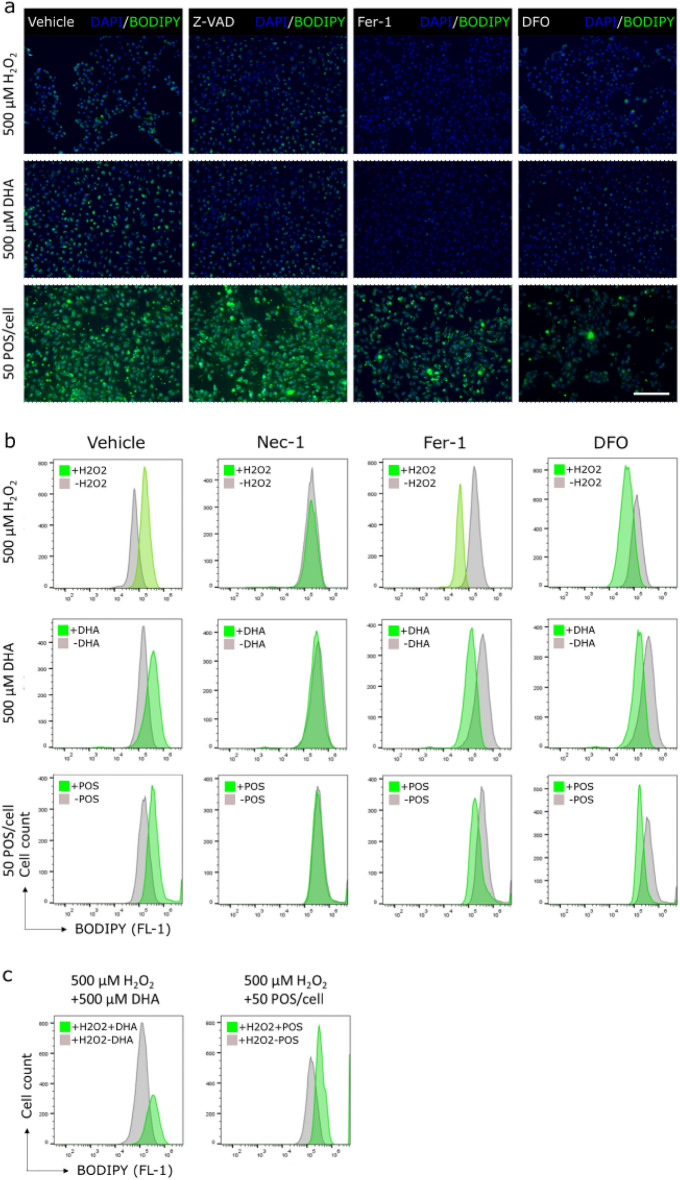
H_2_O_2_-, DHA-, and POS-induced lipid ROS in RPE are reduced by Fer-1 and DFO^36^. ARPE-19 cells were treated with 40 µM Z-VAD, 40 µM Nec-1, or 5 µM Fer-1 24 h or 500 µM DFO 1 h before injury. Subsequent 3-h treatment with 500 µM H_2_O_2_, 500 µM DHA, or 50 POS/cell shows increase of lipid ROS (BODIPY) (**a** and **b**). Pre-treatment with Fer-1 and DFO reduces the amount of lipid ROS after injury. Injury with DHA or POS in addition to H_2_O_2_ increases the amount of lipid ROS compared to H_2_O_2_ alone (**c**). Scale bar equals 200 µm.

Ferroptosis is an iron- and PUFA-dependent form of cell death and is characterised by peroxidation of PUFAs^27^. Phagocytosis of POS, containing high concentrations of the PUFA DHA, could become problematic for RPE under oxidative stress conditions. Involvement of ferroptosis in RPE cell death was therefore investigated by stressing RPE cells with H_2_O_2_, DHA, or POS. Treatment with iron chelator DFO or the ferroptosis inhibitor Fer-1 reduced the amount of lipid ROS caused by H_2_O_2_, DHA, or POS injury (Fig. 1a and b). The necroptosis inhibitor Nec-1 also reduced the amount of lipid ROS but to a more limited extent (Fig. 1b). Necroptosis is a regulated form of necrosis^22^. However, Nec-1 is also known to protect against ferroptosis^35^. In contrast, the apoptosis inhibitor Z-VAD did not reduce the amount of lipid ROS (Fig. 1a). These results show that ferroptosis, and possibly necroptosis, could occur when RPE are stressed with H_2_O_2_, DHA, or POS.

Since DHA is known to enhance RPE survival when oxidatively stressed, the effect of H_2_O_2_ in combination with DHA or POS on RPE were studied by analysing the amount of lipid ROS formed. H_2_O_2_ in combination with DHA showed increased lipid ROS and a similar effect was observed in combination with POS (Fig. 1c). These results indicate that DHA and POS can increase cellular damage during oxidative stress in RPE.

### MLKL is phosphorylated after H_2_O_2_ or POS exposure in RPE

**Figure 2:**
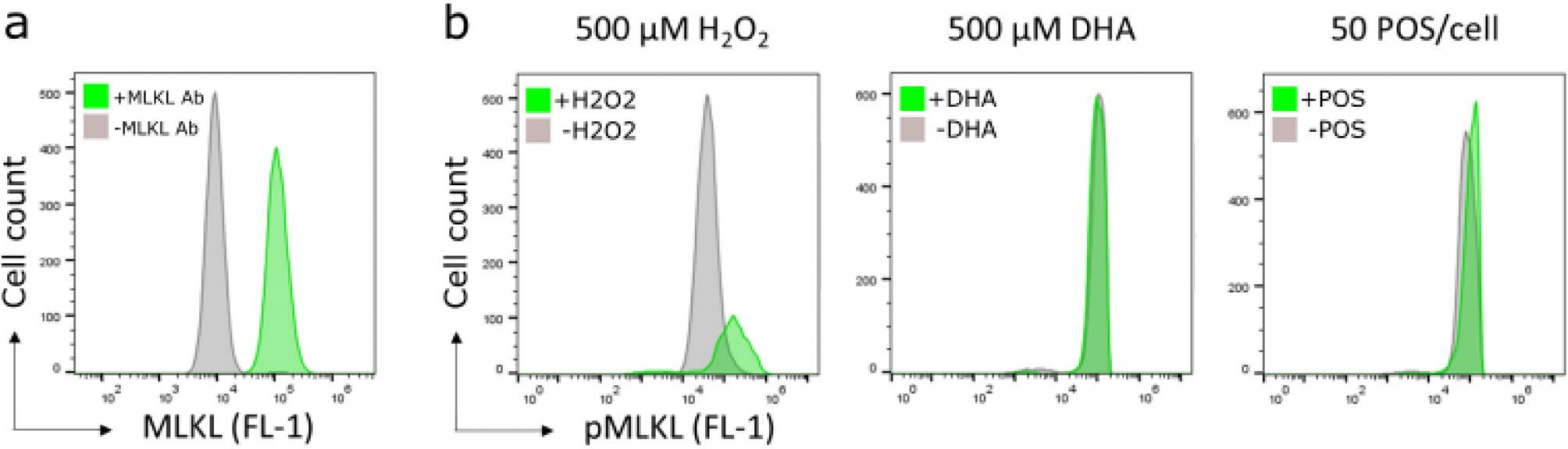
Increase of pMLKL in RPE after H_2_O_2_ or POS exposure^36^. Treatment of ARPE-19 cells with 500 µM H_2_O_2_, but not 500 µM DHA or 50 POS/cell, for 3 h increased phosphorylation of MLKL.

Next, the role of necroptosis was further investigated in RPE stressed with H_2_O_2_, DHA, or POS. MLKL is the effector protein of necroptosis when activated through phosphorylation^32^. RPE were found to express MLKL (Fig. 2a). Moreover, treatment with H_2_O_2_ increased the amount of pMLKL (Fig. 2b) and therefore caused necroptosis. DHA or POS treatment did not increase the amount of pMLKL in RPE and therefore likely do not cause necroptosis without H_2_O_2_. These results indicate that only H_2_O_2_ can induce necroptosis in RPE and that the beneficial effect of Nec-1 on DHA- and POS-induced stress in Fig. 1b seems to be related to the protective effect of Nec-1 on ferroptosis.

### Decreased RPE viability caused by fatty acids during oxidative stress is ameliorated with necroptosis and ferroptosis inhibitors

**Figure 3:**
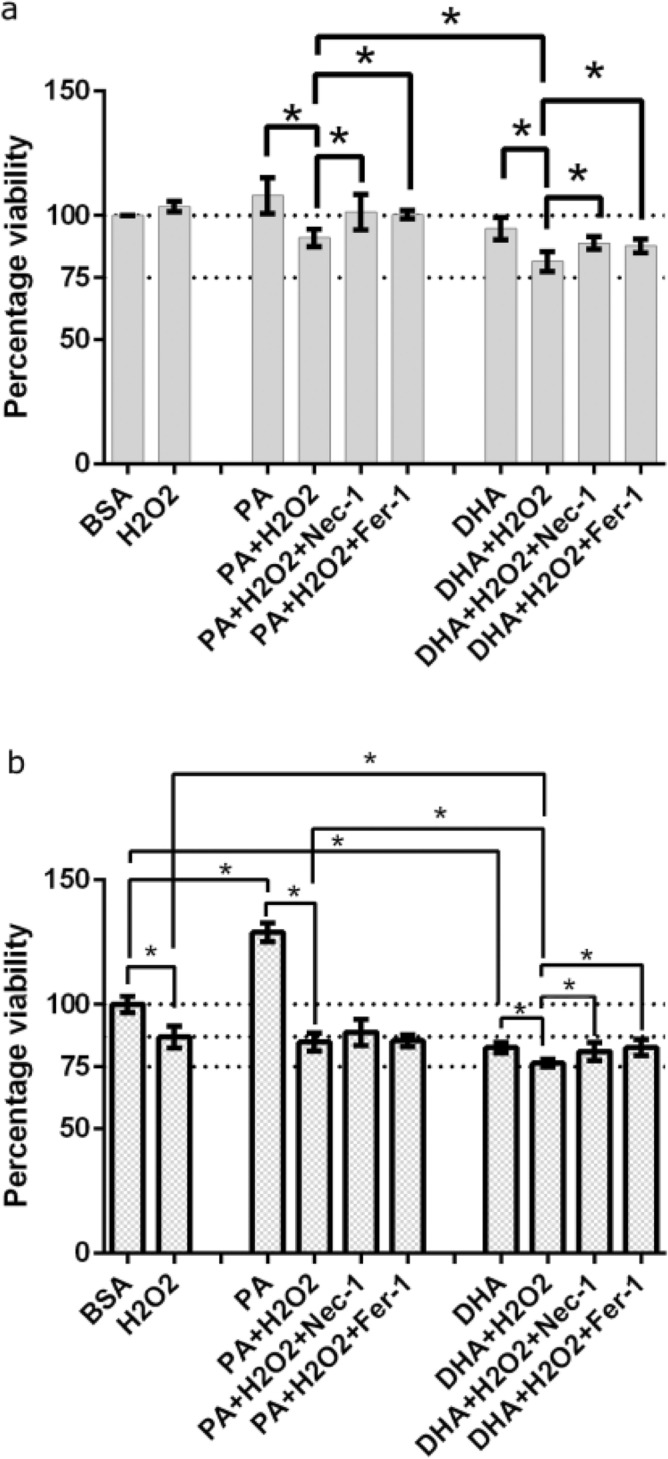
Improved RPE viability with necroptosis and ferroptosis inhibitors after H_2_O_2_ with lipid exposure. The viability of ARPE-19 cells was tested following a 6-h (**a**) or 18-h (**b**) exposure to 500 µM H_2_O_2_, 500 µM PA, 500 µM DHA, the combinations H_2_O_2_ and PA or DHA, or the BSA vector control. Pre-treatment with Nec-1 or Fer-1 helped to improve the viability. Error bars represent SD of three independent experiments. *P ≤ 0.05, determined by Tukey’s test following a one-way ANOVA. BSA = bovine serum albumin; PA = palmitic acid; DHA = docosahexaenoic acid.

To further investigate the role of fatty acids in inducing cellular damage under oxidative stress conditions, RPE were treated with H_2_O_2_ and either PA or DHA. A minimum concentration of H_2_O_2_ was used for inducing oxidative stress (Fig. S2). A 6-h exposure of DHA or PA alone did not reduce cellular viability. However, when combined with H_2_O_2_, RPE cell viability was significantly reduced, with DHA having a more pronounced effect (Fig. 3a). Treatment with either Nec-1 or Fer-1 improved RPE cell viability under these conditions, underlining the role of necroptosis and ferroptosis in RPE cell death during oxidative stress.

When exposed for 18 h, DHA alone significantly reduced RPE cell viability (Fig. 3b). Moreover, a stronger reduction in cell viability was observed when combined with H_2_O_2_. Treatment with Nec-1 and Fer-1 again improved the viability of RPE cells exposed to DHA and H_2_O_2_ for the 18-h exposure. In contrast, RPE cells exposed to PA did not show similar improvements in cell viability with these inhibitors. Notably, 18-h exposure to PA alone showed a marked increase in cell viability, which is likely due to high concentrations of PA can change RPE metabolism (see discussion). Nonetheless, 18-h exposure to PA with H_2_O_2_ lowered the cell viability to the same level as H_2_O_2_ alone and the inhibitors had no positive effect. Together, these results indicate an important role for lipids, and especially PUFAs, during oxidative stress in RPE.

### Transcriptional changes after H_2_O_2_ or DHA exposure in RPE

**Figure 4:**
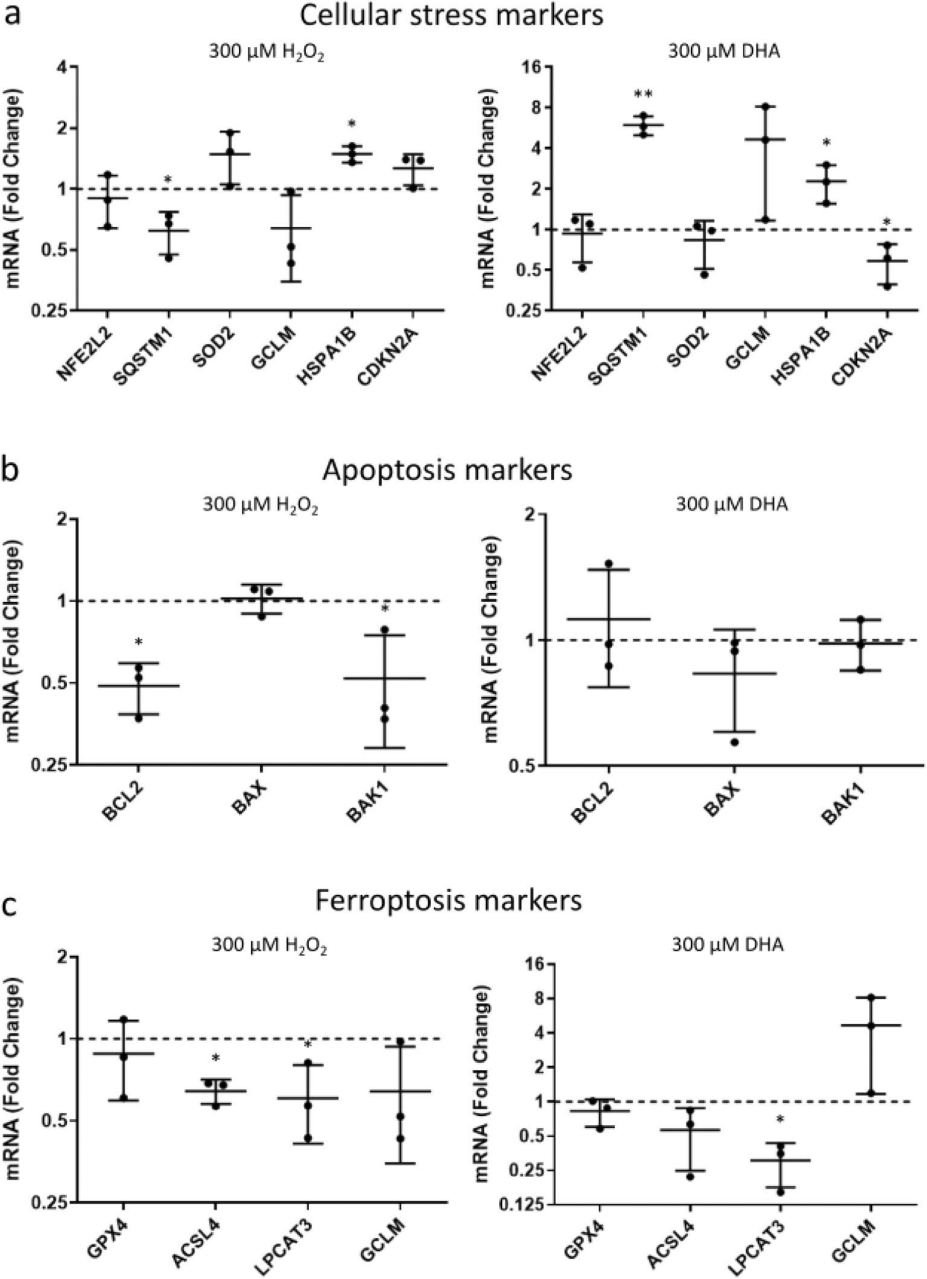
Differential gene expression in RPE exposed to H_2_O_2_ or DHA^36^. ARPE-19 cells were exposed to 300 µM H_2_O_2_ or 300 µM DHA for 24 h. The fold change in mRNA expression was determined of genes related to cellular stress (**a**), apoptosis (**b**), and ferroptosis (**c**). Error bars represent SD of three independent experiments. *P ≤ 0.05 and **P ≤ 0.001, determined by *t*-tests on ΔCt values relative to corresponding controls.

In order to investigate the molecular pathways involved, the transcriptional changes between RPE cells injured with H_2_O_2_ or DHA were studied. First, differential expression of common cellular stress markers was observed. SQSTM1, also known as p62, is a multifunctional protein involved in cellular processes including autophagy and stress. *SQSTM1* expression in RPE was significantly downregulated upon H_2_O_2_ exposure (Fig. 4a). In contrast, DHA caused a significant upregulation of *SQSTM1* expression. HSPA1B, also known as Hsp70, is involved in a wide range of cellular processes, including protection of the proteome against stress. *HSPA1B* expression was significantly upregulated by both H_2_O_2_ and DHA exposure (Fig. 4a). CDKN2A, also known as p16, is involved in regulating the cell cycle and is regarded as a marker of cellular senescence. *CDKN2A* expression was significantly downregulated by DHA exposure, but not H_2_O_2_ exposure (Fig. 4a). These results highlight the markedly different response of RPE towards different forms of stress.

Cells can respond to stress in various ways, from activating pathways promoting survival, to triggering specific cell death pathways. To investigate if apoptotic signalling was induced, the expression of certain apoptosis markers was measured following H_2_O_2_ or DHA exposure. BCL2, an important apoptosis regulator preventing apoptotic cell death, was significantly downregulated in RPE exposed to H_2_O_2_ (Fig. 4b). However, the expression of proapoptotic marker *BAX* did not significantly change and moreover the expression of proapoptotic marker *BAK1* was significantly downregulated (Fig. 4b). *BCL2*, *BAX* and *BAK1* expression did not significantly change in RPE exposed to DHA (Fig. 4b). These results again suggest that apoptosis does not play a role in H_2_O_2_-induced cell death and that high DHA-exposure also does not lead to apoptosis in RPE.

Expression differences of ferroptosis-related genes was also assessed in H_2_O_2_- or DHA-mediated cell death in RPE. Interestingly, the expression of *acyl-CoA synthetase long-chain family member 4* (*ACSL4*) and *lysophosphatidylcholine acyltransferase 3* (*LPCAT3*) were significantly downregulated in RPE treated with H_2_O_2_ (Fig. 4c). In contrast, in DHA treated RPE only *LPCAT3* expression was significantly downregulated (Fig. 4c). ACSL4 and LPCAT3 are important regulators of ferroptosis. Other important genes involved in ferroptosis such as *GPX4* and *GCLM* did not significantly change in expression in RPE injured with H_2_O_2_ or DHA. Together, these results indicate that when exposed to H_2_O_2_ or DHA, RPE cells modulate the expression of genes important to induce ferroptosis, in order to prevent more ferroptotic cell death.

### Decreased expression of ACSL4 after H_2_O_2_ and DHA exposure in RPE

**Figure 5:**
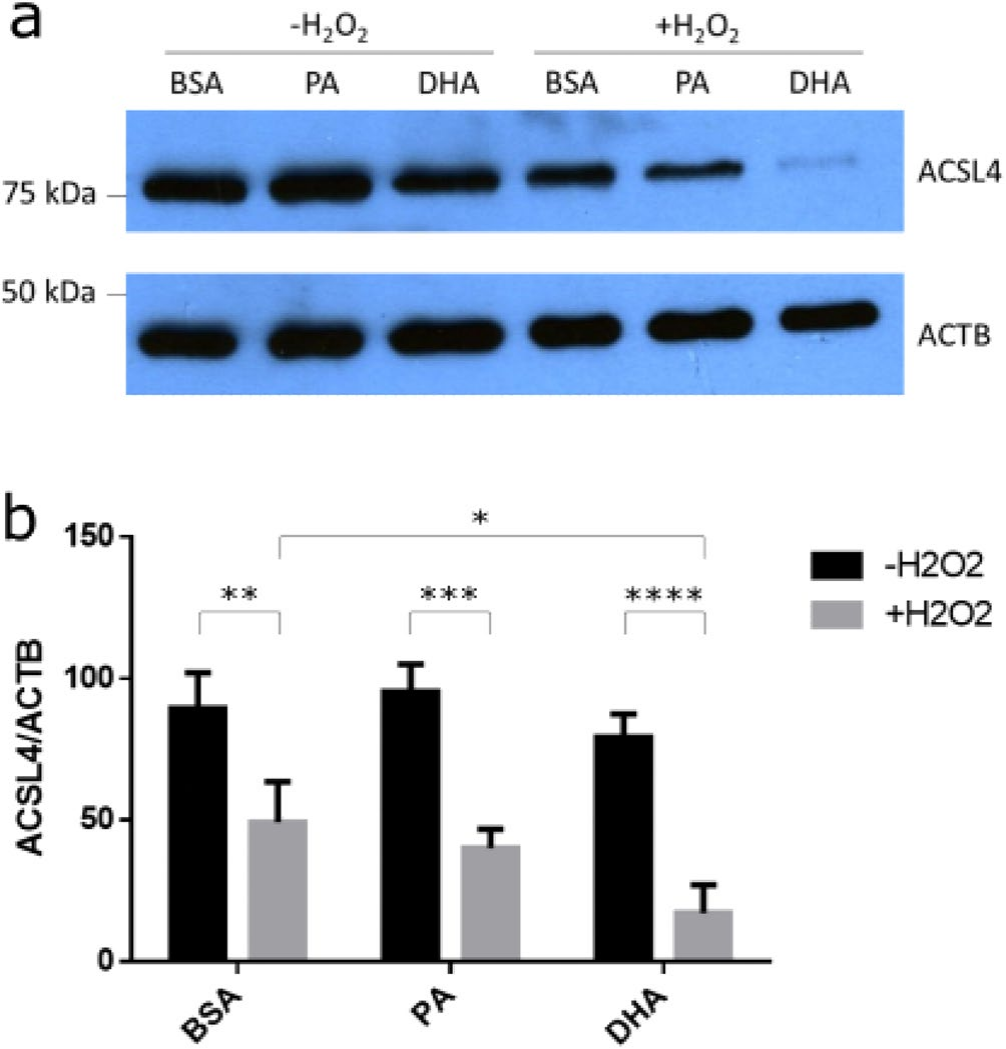
ACSL4 is downregulated in RPE after H_2_O_2_ and DHA exposure^36^. ARPE-19 cells were exposed to 500 µM H_2_O_2_, 500 µM PA, 500 µM DHA or the BSA vector control for 6 h. Representative blot out of three depicting ACSL4 expression changes to H_2_O_2_ and fatty acids (**a**). Densitometric analysis (**b**). Error bars represent SD of three independent experiments. *P ≤ 0.05, **P ≤ 0.01, ***P ≤ 0.001, ****P ≤ 0.0001, determined by Šidák’s multiple comparisons test following a two-way ANOVA. BSA = bovine serum albumin; PA = palmitic acid; DHA = docosahexaenoic acid. Original blots are presented in Fig. S3.

To further investigate the observed downregulation of the *ACSL4* gene in H_2_O_2_-treated RPE cells, the protein expression was analyzed. Again, H_2_O_2_ significantly reduced the expression of ACSL4 (Fig. 5a compare lanes 1 and 4 and Fig. 5b compare bars 1 and 2) and DHA did not significantly reduce the expression of ACSL4 (Fig. 5a compare lanes 1 and 3 and Fig. 5b compare bars 1 and 5), consistent with the findings in Fig. 4c. Exposure of the RPE cells to the most common saturated fatty acid in POS, PA, showed a similar result as DHA (Fig. 5a compare lanes 1 and 2 and Fig. 5b compare bars 1 and 3).

RPE cells were also exposed to a combination of H_2_O_2_ and either PA or DHA to determine how the combined exposure of fatty acids and H_2_O_2_ could affect ACSL4 expression. H_2_O_2_ significantly reduced the ACSL4 protein levels and the addition of DHA, but not PA, exacerbated this effect significantly more (Fig. 5a compare lanes 4, 5, and 6, and Fig. 5b compare bars 2, 4, and 6). These results indicate that DHA has a specifically negative effect on the expression of ACSL4 under oxidative stress conditions.

## Discussion

AMD is a multifactorial disease and oxidative stress is known to play an important role in the initiation and progression of this disease. Still much is unknown about how oxidative stress contributes to AMD pathology. Elucidating how oxidative stress affects RPE, the cell type severely affected in AMD, could contribute to our understanding of AMD initiation and progression. Here, we show that oxidative stress causes both necroptosis and ferroptosis, while addition of the PUFA DHA can worsen this effect.

Several studies have reported numerous health benefits of dietary DHA, such as supporting fetal development, preventing preterm birth, cardiovascular disease prevention, and improving cognitive function^37^. Although DHA is essential for normal functioning of the retina and is known for its neuroprotective functions, DHA can also play an important role in mediating cell death. Approximately 50% of the phospholipids in the vertebrate rod photoreceptors contain DHA^38^. RPE phagocytose POS containing this high concentration of DHA. DHA-containing phospholipids are reservoirs for docosanoids, potent bioactive mediators. The docosanoid neuroprotection D1 (NPD1) forms after oxidative stress and promotes RPE survival^20^. However, increasing dietary DHA uptake has shown to improve intermediate AMD, but not advanced AMD^39^. Moreover, with age, phagocytosis of POS can lead to lipid accumulation in RPE, leading to ROS production^40^. This is likely caused by carboxyethylpyrrole (CEP) protein adducts, which are formed by an oxidation product unique to DHA^41^. CEP adducts are found to be more abundant in AMD retinal tissue, specifically in the RPE, Bruch’s membrane, and choroid^42^. In addition, CEP adducts are also found in AMD drusen^42^ and in lipofuscin^43^. This indicates that DHA peroxidation is increased and that ferroptosis could be a common form of cell death in AMD. CEP adducts have been shown to stimulate angiogenesis^44,45^, thus possibly contributing to neovascularization in geographic atrophy AMD. Moreover, mice immunised with CEP adducts develop a geographic atrophy AMD-like phenotype, indicating a role for CEP adducts in the aetiology of AMD^46^. CEP adducts were also found to be elevated in the plasma of patients with AMD^41^. CEP adducts could therefore play a role in AMD pathology and possibly function as a biomarker for AMD. It is therefore possible that in a disease setting like AMD, with prolonged oxidative stress in combination with high concentrations of DHA, the negative consequences of the formation of CEP adducts outweigh the positive benefits of the formation of NPD1, leading to an overall negative impact on cell viability by promoting necroptotic and ferroptotic cell death.

Ferroptosis is characterised by peroxidised PUFAs and is iron-dependent. DHA has been shown to efficiently promote ferroptosis before when incorporated into cellular membranes^28^. The amount of lipid ROS increased in RPE injured with H_2_O_2_, DHA, and POS (Fig. 1). Lipid ROS generated in this way was reduced with Fer-1, DFO, and Nec-1, further confirming a role for ferroptosis in these types of RPE injuries. Nec-1 is known to also have a protective effect against ferroptosis, but the mechanism is not yet known^35^. Increased iron accumulation is found within the retina of AMD patients^47,48^, which is thought to promote oxidative stress through the Fenton reaction. Iron was also recently found to promote oxidative cell death caused by bisretinoids in the retina^49^, the precursor of lipofuscin. Therefore, iron accumulation could play an important role in the pathology of AMD. Moreover, it is known that iron chelation can protect RPE^50^. It is therefore possible that in a disease setting like AMD, DFO could at least reduce RPE ferroptosis. Furthermore, addition of DHA or POS to H_2_O_2_-mediated oxidative injury increased the amount of lipid ROS production in RPE (Fig. 1c). This indicates that DHA can increase the amount of ferroptotic cell death of RPE during oxidative stress.

Injury of RPE with H_2_O_2_ also caused an increased phosphorylation of S358 of MLKL (Fig. 2b). This specific phosphorylation of MLKL is necessary for necroptosis^32^. However, this was not found in RPE treated with only DHA or POS. These results show that necroptosis plays an important role in H_2_O_2_-induced oxidative stress in RPE, in addition to ferroptosis, but necroptosis does not seem to play a role in PUFA-induced stress.

RPE treated with fatty acids and subsequently injured with H_2_O_2_ resulted in decreased cell viability compared to injury with H_2_O_2_ alone (Fig. 3). When exposed for 6 h to H_2_O_2_, Fer-1 and Nec-1 could rescue additional stress caused by PA and DHA (Fig. 3a). However, under prolonged oxidative stress, when the cells are exposed for 18 h to H_2_O_2_, Fer-1 and Nec-1 could not rescue the cells additionally stressed with PA (Fig. 3b). Prolonged PA exposure does increase the cell viability compared to the BSA control, but not in the presence of H_2_O_2_. This indicates that at least in the case of prolonged PA exposure there are other cellular processes counteracting the cell death mechanisms. For example, PA has been reported to induce epithelial-mesenchymal transition (EMT) in ARPE-19 cells^51^. Additionally, RPE cells are reported to have the ability to oxidize PA, generating acetyl CoA and β-hydroxybutyrate, a process that increases the mitochondrial oxygen consumption rate^52^. This increased mitochondrial metabolic activity or proliferation, possibly due to EMT, could be responsible for the elevated cellular viability observed with PA at 18 h. However, 18-h treatment with the PUFA DHA showed a significantly more decreased cell viability, indicating that peroxidation of DHA could exacerbate oxidative stress in RPE. Importantly, subsequent treatment with necroptosis or ferroptosis inhibitors significantly alleviated this effect. This indicates that oxidative stress in RPE can lead to both necroptosis and ferroptosis, and that this is influenced by DHA.

A differential transcriptomic response was found in RPE injured with H_2_O_2_ or DHA (Fig. 4), with a differential expression of common cellular stress markers (Fig. 4a). SQSTM1, also known as p62, was upregulated in RPE exposed to DHA. This is consistent with previous findings where DHA induces *SQSTM1* expression and thereby increases autophagy^53^. Important genes involved in apoptosis did not change expression necessary for apoptosis in RPE exposed to H_2_O_2_ or DHA (Fig. 4b), suggesting that RPE use an alternate mode of cell death when oxidatively stressed. The levels of the antioxidant GSH decreases with age and this has been linked to AMD^6^. The important ferroptosis inhibitor GPX4 is GSH-dependent. However, oxidative stress caused by H_2_O_2_ or DHA injury did not change the expression of *GPX4* or *GCLM* in RPE (Fig. 4c). Together, these results show a differential transcriptomic response between RPE injured with H_2_O_2_ compared to DHA in terms of the expression of stress-related genes, but a similar response in terms of apoptosis-related genes and ferroptosis-related genes.

Interestingly, *ACSL4* was found to be downregulated in expression in RPE injured with H_2_O_2_ (Fig. 4c). ACSL4 was also found to be downregulated in protein expression in RPE injured with H_2_O_2_ and this was exacerbated when DHA was added under oxidative stress (Fig. 5). The most common saturated fatty acid in POS, PA, did not show the same effect. Additionally, *LPCAT3* was found to be downregulated in RPE injured with H_2_O_2_ or DHA (Fig. 4c). ACSL4 and LPCAT3 are involved in the execution of ferroptosis^54^. ACSL4 catalyses the activation of PUFAs by esterification, producing acyl-CoA. Acyl-CoA is an intermediate in various metabolic pathways, including phospholipid production for membrane biogenesis. ACSL4 is found to change lipid composition which affects ferroptosis sensitivity^28,55^. ACSL4 promotes the incorporation of omega-6 PUFAs in cellular membranes, which makes cells more sensitive to ferroptosis. ACSL4 has been proposed as a biomarker for ferroptosis and inhibition of ACSL4 also promotes necroptosis^56^. Downregulation of ACSL4 in response to H_2_O_2_ and downregulation of LPCAT3 in response to H_2_O_2_ or DHA could indicate that RPE have a defensive response towards oxidative stress and PUFAs to prevent ferroptosis and induce necroptosis. However, the concentrations that were used in this study could have been too high or too prolonged to prevent ferroptosis from occurring. Similarly, this defence mechanism could also be overwhelmed in a disease setting like AMD.

It is thought that necroptosis and ferroptosis can compensate each other when either one is inhibited^56^. H_2_O_2_-induced oxidative stress in RPE in this study caused both increased phosphorylation of MLKL and increased lipid ROS formation. However, the important regulators ACSL4 and LPCAT3 were found to be downregulated after H_2_O_2_ injury and the additional presence of excess DHA decreased the expression of ACSL4 even more. Inhibition of ACSL4 is known to promote necroptosis^56^. This shows that there is a complex regulation of the different cell death pathways in response to oxidative stress that may be interconnected. The proclivity of RPE cells to undergo cell death through necroptosis and ferroptosis after oxidative injury have both been found before^22,24^ but a role for DHA in this process has not been fully established in RPE. Ferroptosis in particular seems a logical form of cell death in RPE during AMD because of the accumulation of DHA through phagocytosis of POS and the increased oxidative stress. Here, we showed that the detrimental effects of H_2_O_2_-induced oxidative stress in RPE was exacerbated with DHA which led to both necroptosis and ferroptosis.

### Supplementary Information

Supplementary Information.

## Data Availability

The datasets used and/or analyzed during the current study are available from the corresponding author on reasonable request.
