# Supplementary material for: Oxidative stress and docosahexaenoic acid injury lead to increased necroptosis and ferroptosis in retinal pigment epithelium
Source: Sci Rep. 2023 Nov 30;13:21143. doi: 10.1038/s41598-023-47721-5 (PMC10689458; doi:10.1038/s41598-023-47721-5)
Supplement: Supplementary file 1 — Supplementary Information. [file 41598_2023_47721_MOESM1_ESM.docx]

**Supplementary Information**

**Table S1 | qPCR primer sequences.**

| Name | Accession | Forward (5`-3`) | Reverse (5`-3`) |
| --- | --- | --- | --- |
| 18S | M10098 | * | * |
| ACSL4 | NM_004458 | ATTCTTCTCCGCTTACACTCTCT | CTCTTGGACTTTGCTCATAACATTC |
| ADIPOR1 | NM_00129055 | CTTCAAGAGCATCTTCCGCATT | GGTCTGAGCATGGTCAAGATTC |
| BAK1 | NM_001188 | GCCAAGGTCCTGCTCAACT | CACCCCAAGCCCAGAATCC |
| BAX | NM_138761 | ATGGAGCTGCAGAGGATGAT | CAGTTGAAGTTGCCGTCAGA |
| BCL2 | NM_000633 | GAGGTCACGGGGGCTAATT | GAGGCTGGGCACATTTACTG |
| C1QTNF5 | NM_015645.4 | GGCAAGTTCACCTGCCAGG | TCGCCATTCTTCACCAGATCAA |
| CDKN2A | NM_000077 | ATGTCCTGCCTTTTAACGTAGATA | CTCACTCCAGAAAACTCCAACA |
| GCLM | NM_002061.3 | GGAATTATCAAATCAAAAGGCTACATT | TTTTTACACATCTCAATTTTCTCTCAT |
| GPX4 | NM_002085 | AGTAACGAAGAGATCAAAGAGTTC | CCTTGCCCTTGGGTTGG |
| HSPA1B | NM_005346.4 | GCGTGATGACTGCCCTGAT | GTTGTCGGAGTAGGTGGTGAA |
| LPCAT3 | NM_005768.5 | CAGAGCTGATTGACATACCAGGA | AGGAGATAGTCTTCTGTGATGTGG |
| NFE2L2 | NM_006164.4 | CCCAGCACATCCAGTCAGA | CAGTCATCAAAGTACAAAGCATCT |
| PPARA | NM_00100192 | TCATCACGGACACGCTTTCA | TCCCCGCAGATTCTACATTCG |
| PPARG | NM_138711 | GAATAAAGATGGGGTTCTCATATCC | AACTTCACAGCAAACTCAAACTT |
| PRKAA1 | NM_006251 | GCATCCTCATATAATTAAACTGTACCA | TTCATCCAGCCTTCCATTCTTAC |
| SOD2 | NM_000636.3 | CGACCTGCCCTACGACTAC | AACGCCTCCTGGTACTTCTC |
| SQSTM1 | NM_003900.4 | ACCATCCAGTATTCAAAGCATCC | AAGAGGGGCACGCAGAAG |

*Primerdesign proprietary sequence


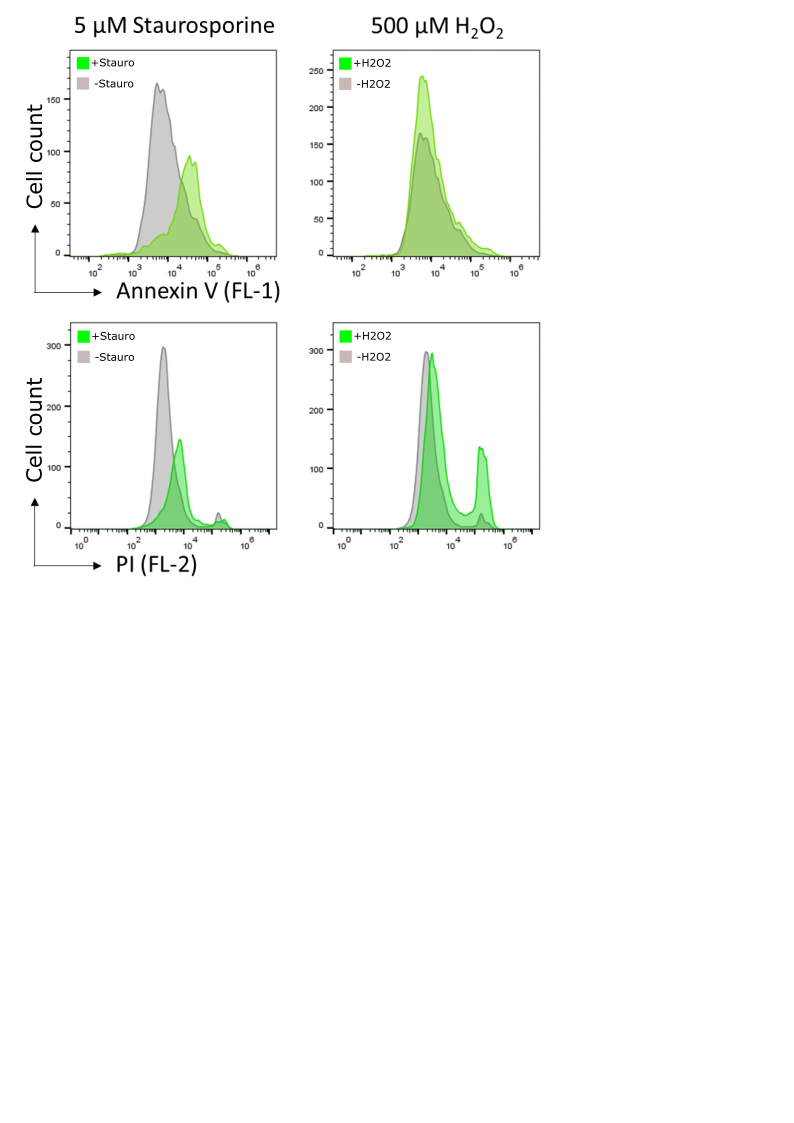


**Figure S1 | H_2_O_2_ exposure leads to necrotic and not apoptotic cell death in RPE.** ARPE-19 cells were treated with 5 µM staurosporine or 500 µM H_2_O_2_ for 3 hours. Staurosporine treatment, a positive control for apoptosis-induction, but not H_2_O_2_ exposure leads to increased Annexin V staining, a marker for apoptosis. H_2_O_2_, but not staurosporine exposure leads to increased PI staining, a marker for necrosis.


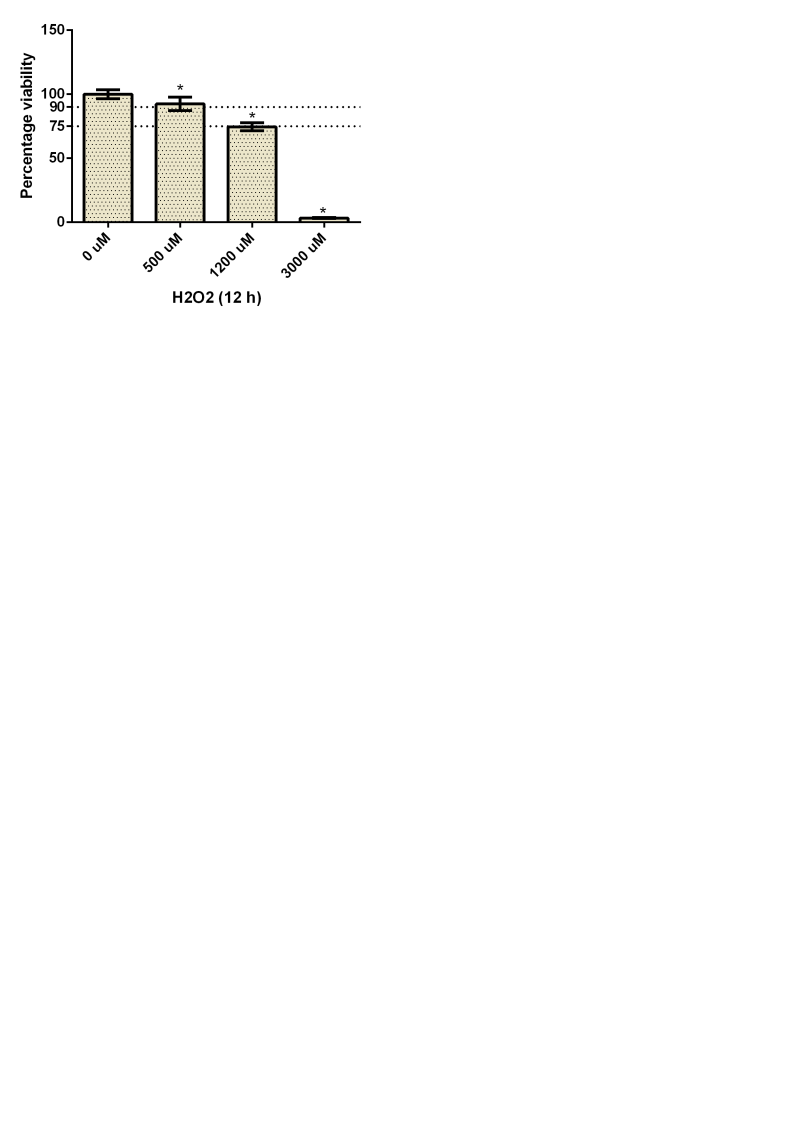


**Figure S2 | Viability of ARPE-19 cells on exposure to H_2_O_2_.** Viability of ARPE-19 cells on exposure to different concentrations of H_2_O_2_ for 12 hours quantified by MTT assay. Error bars represent SD of three independent experiments. *P≤0.05, determined by Dunnett's test following a one-way ANOVA.


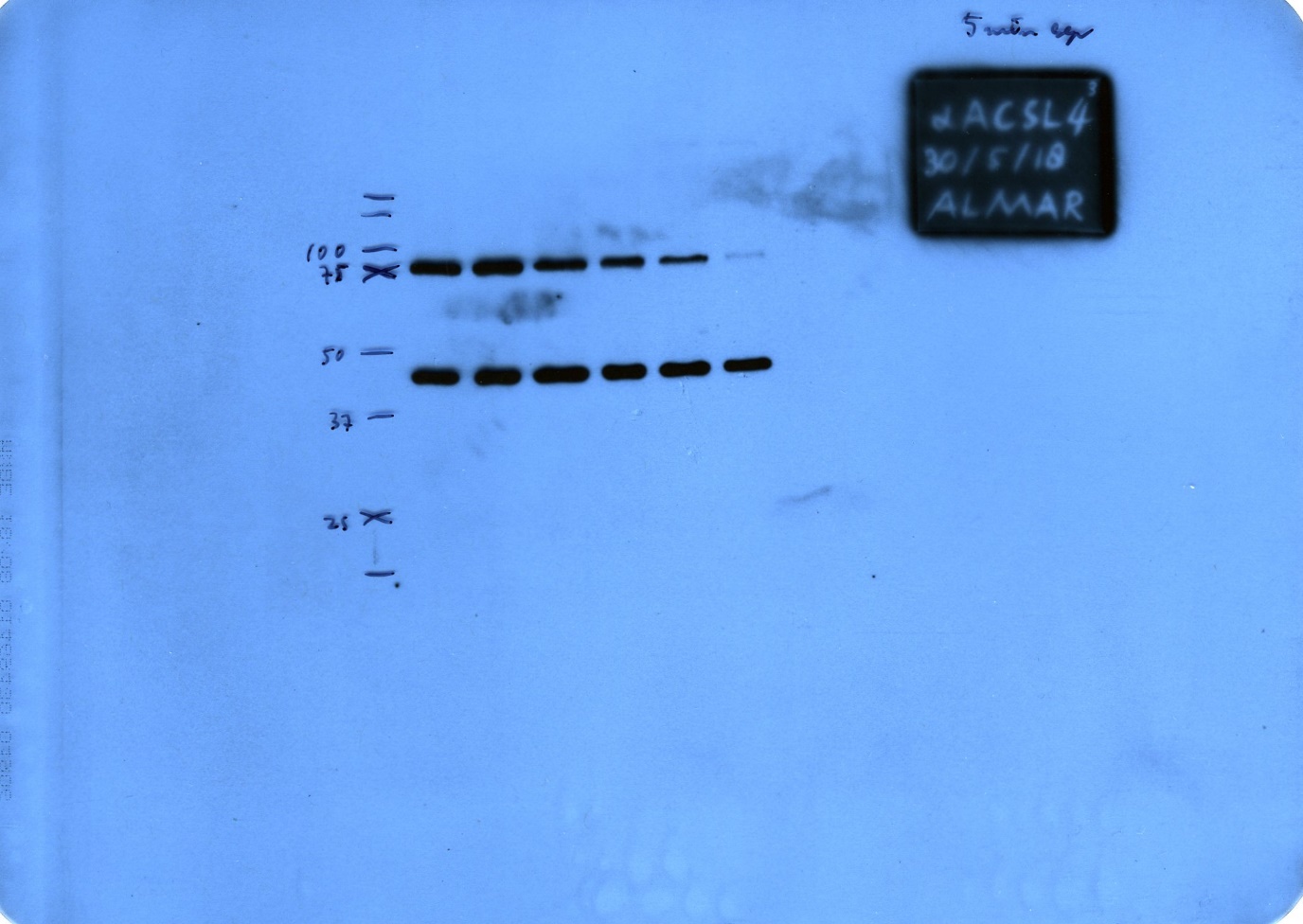

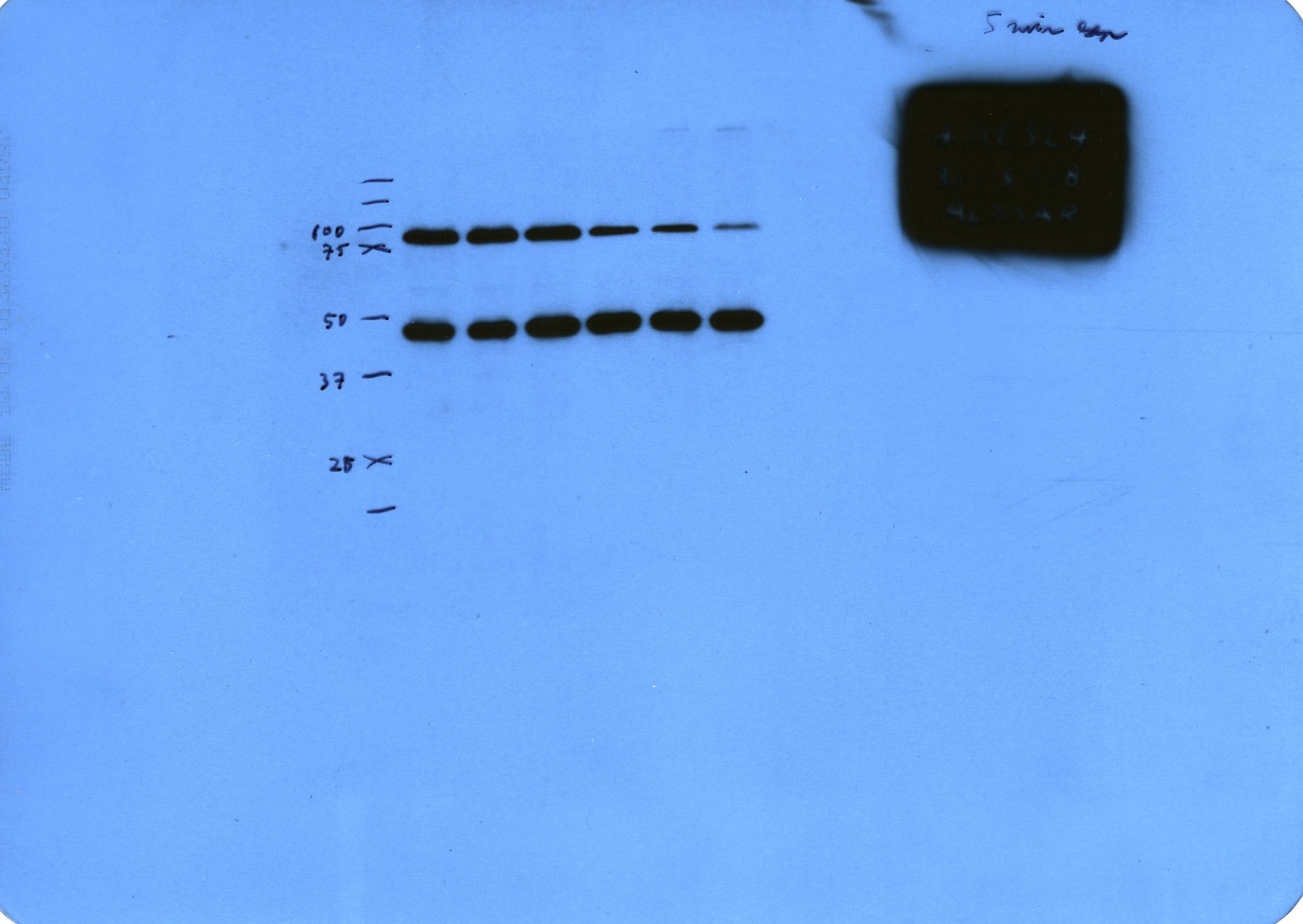

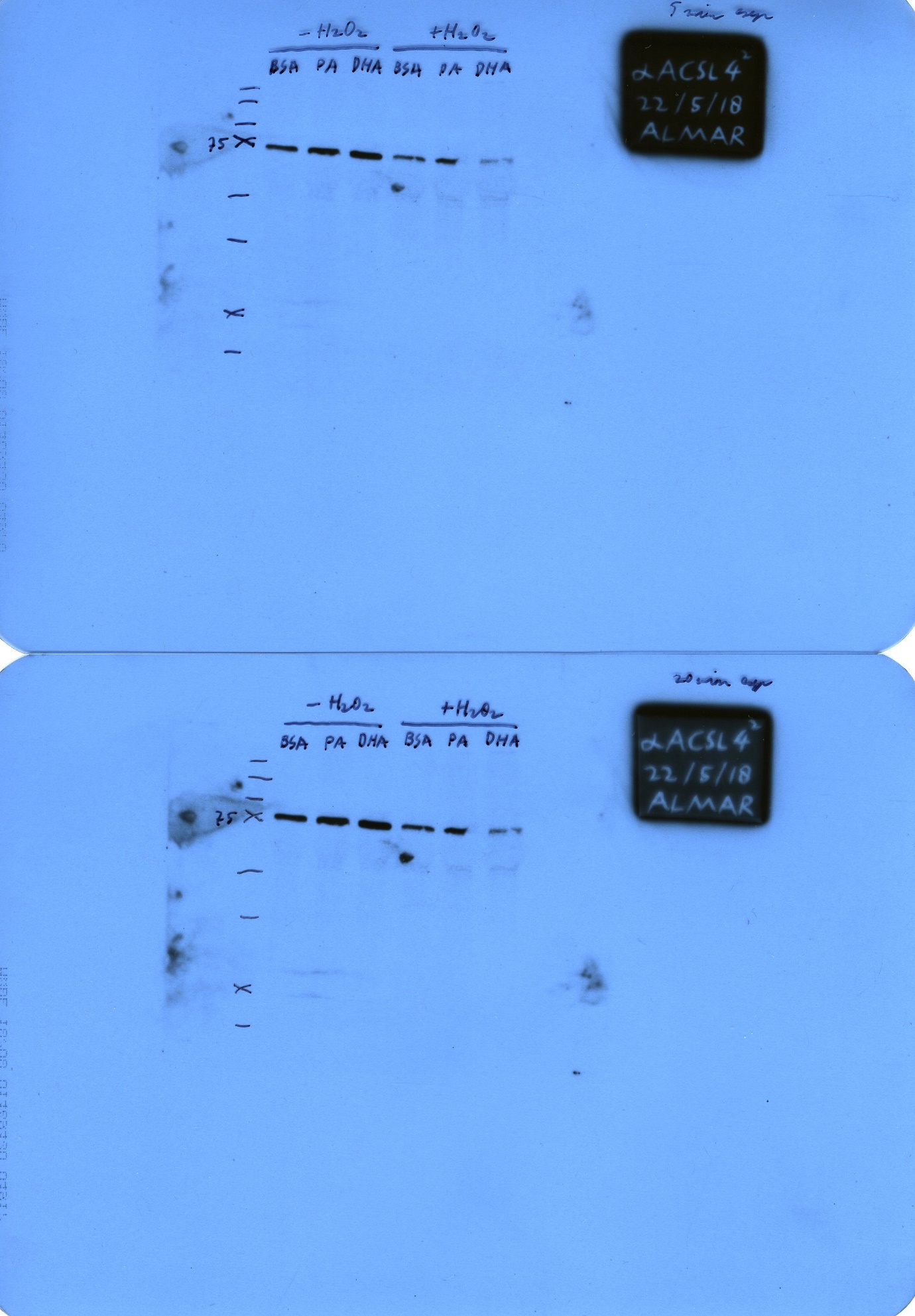

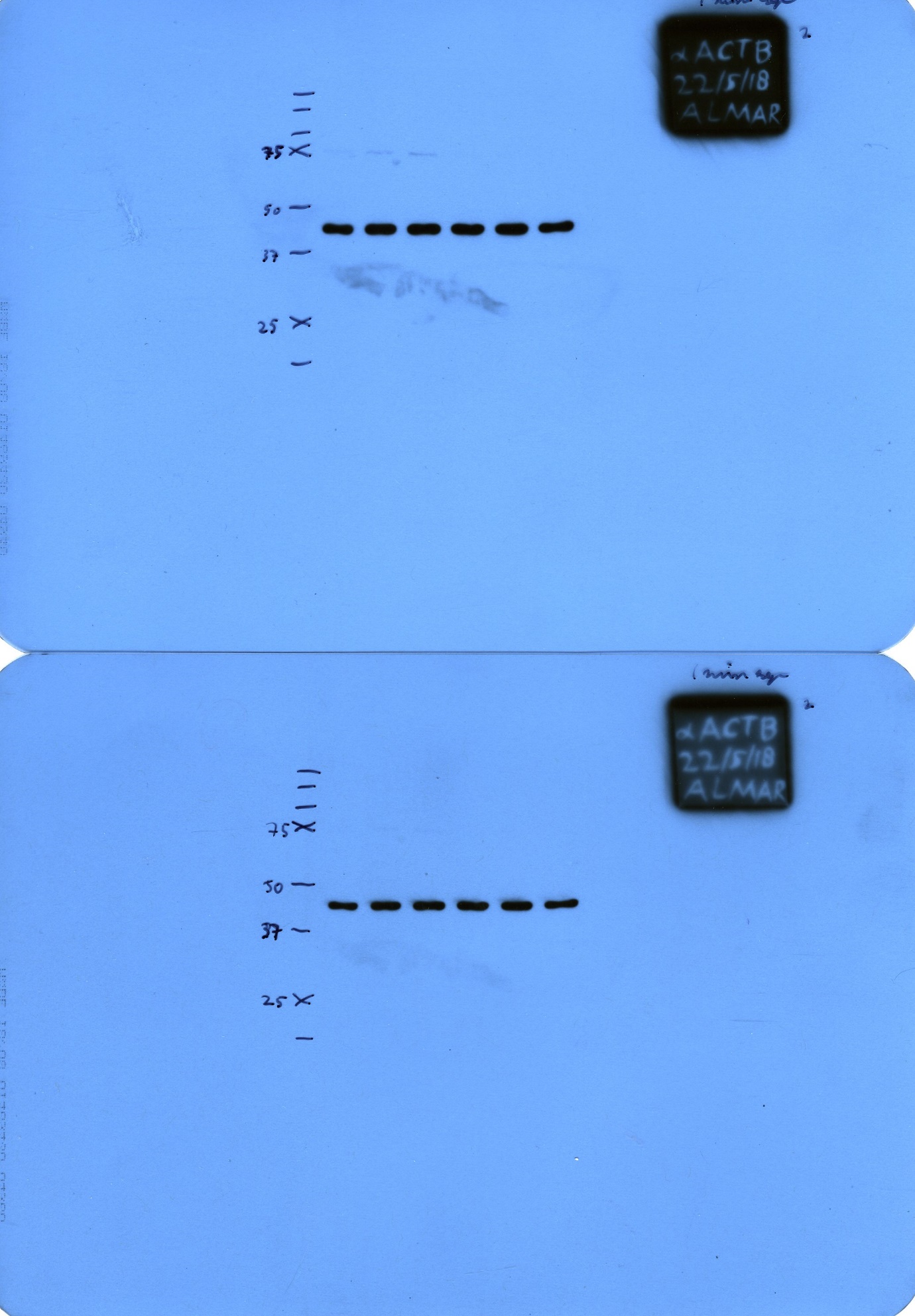


**Figure S3 | ACSL4 is downregulated in RPE after H_2_O_2_ and DHA exposure.** Original uncropped blot for Figure 5, including the blots used for the quantification, and including different exposure times for some of them.
